# A semi-labelled dataset for fault detection in air handling units from a large-scale office

**DOI:** 10.1016/j.dib.2024.110956

**Published:** 2024-09-21

**Authors:** Seunghyeon Wang, Ikchul Eum, Sangkyun Park, Jaejun Kim

**Affiliations:** Department of Architectural Engineering, Hanyang University, Seungdong-Gu, Seoul 133791, Republic of Korea

**Keywords:** HVAC system, Air handling units, Fault detection, Fault diagnosis, Deep learning, Machine learning

## Abstract

Fault detection and diagnosis (FDD) in Air Handling Units (AHUs) ensure building functions such as energy efficiency and occupant comfort by quickly identifying and diagnosing faults. Combining deep learning with FDD has demonstrated high generalization ability in this field. To develop deep learning models, this research constructed a dataset sourced from real data collected from a large-scale office in South Korea. The raw AHU data were extracted from the Building Management System (BMS) at 1-h intervals, spanning from November 2023 to May 2024. The dataset was partially labeled by annotation experts, categorizing the data into six types: normal condition, supply fan fault, total heating pump fault, return air temperature sensor fault, supply air Temperature sensor fault, and valve position fault. Additionally, semi-supervised learning methods were applied as an application example using this constructed dataset. The main contributions of this dataset to the field are twofold. First, it represents a unique dataset sourced from the real operational data of a large-scale office, which is currently non-existent in this domain. Second, the dataset's expert labeling adds significant value by ensuring accurate fault classification. Therefore, we hope that this dataset will encourage the development of robust FDD techniques that are more suitable for real-world applications.

Specifications Table

**Table:** 

Subject	Mechanical Engineering, Environmental Engineering, Artificial Intelligence
Specific subject area	Fault diagnosis of AHUs in HVAC
Type of data	Table
Data collection	Data was extracted from a large-scale office through 20 different AHUs in the HVAC system. Eleven independent variables related to the AHUs were collected: set point temperature, return temperature, supply air temperature, supply fan speed, valve position, heating supply temperature 1, heating supply temperature 2, total heating pump, heating pump 1, heating pump 2, and backup heating pump. The data was then labeled with the corresponding classification types: normal condition, supply fan fault, total heating pump fault, return air temperature sensor fault, supply air temperature sensor fault, and valve position fault.
Data source location	Country: South Korea, Cities: Gwacheon
Data accessibility	Repository name: FigShare Data identification number: https://doi.org/10.6084/m9.figshare.25909813.v1 Direct URL to data: https://figshare.com/articles/dataset/A_semi-labelled_dataset_for_fault_detection_in_air_handling_units_from_a_large-scale_office/25909813/1
Related research article	None

## 1 Values of the Data

• This dataset addresses the lack of AHU in HVAC fault data from real operational data in larger scale offices.
• Both industrial practitioners and those from the academic community may benefit from this dataset.
• As part of the dataset is labeled, semi-supervised learning based on a data-driven approach is recommended to investigate the importance of trends and patterns in the data in relation to detecting and diagnosing faults.
• This dataset can be used to benchmark new AHU fault detection algorithms against real-world data, providing a reliable standard for evaluating performance.
• Researchers can utilize this dataset to develop and validate advanced machine learning algorithms for AHU fault detection and diagnosis, enhancing the accuracy and reliability of these systems.

## 2 Background

The impacts of AHU (Air Handling Unit) faults on office buildings have been extensively studied, as faulty AHUs can affect occupant comfort, air quality, and energy efficiency [1]. Fault Detection and Diagnosis (FDD) in AHU systems involves monitoring components like sensors and controllers to quickly identify issues [2]. Data-driven methods such as machine learning and deep learning, leveraging extensive annotated AHU data, have shown high accuracy. However, most FDD model research relies on simulation data, which does not accurately reflect real-world conditions, leading to low accuracy in many studies [3]. Despite the potential benefits, collecting faults in AHUs is challenging. Accurately labeling data for fault detection requires domain expertise to distinguish between normal operation and actual faults. Additionally, protecting the collected data from unauthorized access, breaches, and cyber-attacks is critical. There is also no publicly available raw and labeled data for real operational faults in AHUs, highlighting a significant gap in prior research. An aim of this article is to provide a dataset sourced from real operational data in a large-scale office to encourage the development of robust techniques for distinguishing faults in AHUs. This dataset can be used for future research to translate simulation results into real-world applications.

## 3 Data Description

The data for independent variables related to AHU faults consists of eleven variables: set point temperature, return temperature, supply air temperature, supply fan, valve position, heating supply temperature 1, heating supply temperature 2, total heating pump, heating pump 1, heating pump 2, and backup heating pump. This information was collected through various sensor readings installed at strategic points across 20 different AHUs. Each sensor continuously monitors and records the relevant variable, providing a detailed snapshot of the AHU's operational status.

Return air temperature and supply air temperature are measured using sensors placed in the return and supply air ducts, respectively. Additionally, the heating supply temperature, recorded at two different points, is also monitored using specialized temperature sensors placed within the heating supply lines. Fan operation is another critical aspect of AHU performance, and it is monitored using fan sensors. These sensors, which can be either current sensors or airflow sensors, provide real-time data on the operational status and performance of the supply fan. Similarly, valve positions, which indicate the degree to which a valve is open or closed, are measured using position sensors. Pumps, integral to the heating supply, are monitored using a combination of current sensors and flow meters. These sensors track the operational status and flow rate of the total heating pump, individual heating pumps, and any backup heating pumps.

**Fig. 1:**
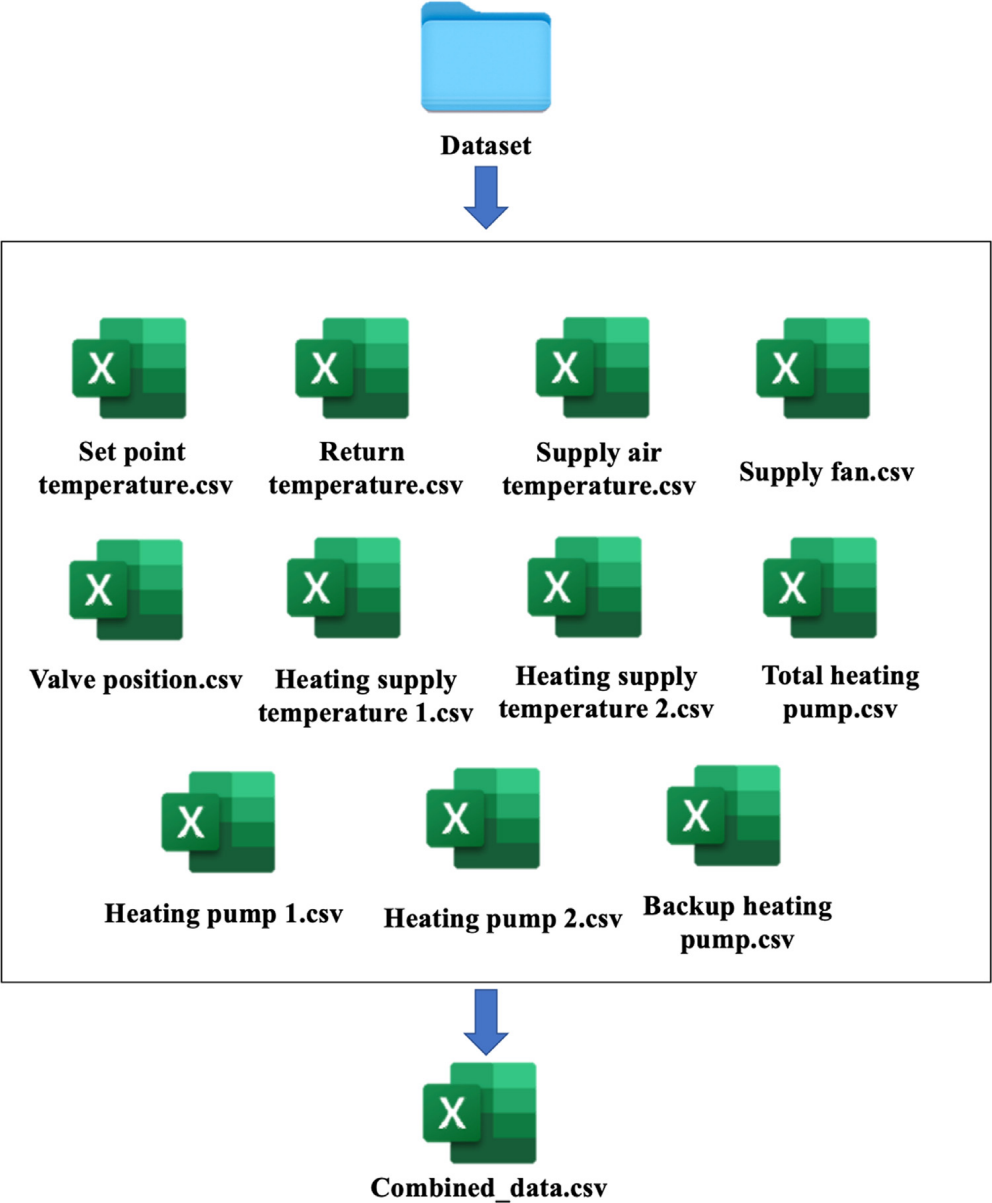
Structure of the dataset.

All these sensors are integrated into the BMS, allowing for continuous monitoring and control of the AHU operation. Initially, the data for each independent variable was stored separately in CSV format. These CSV files were loaded directly from the BMS, which is responsible for managing and monitoring the building's HVAC systems. Fig. 1 illustrates how these CSV files are organized.

After the initial data collection, the separate CSV files were combined into a single comprehensive dataset. The code for processing and combining each file can be accessed here [4]. To facilitate analysis, the raw data was processed and aggregated into one-hour intervals. Instances with missing data were removed if more than one independent variable was missing. Additionally, each data point in the dataset was labeled and categorized into one of six distinct types:

• Normal condition: 40,052
• Supply fan fault: 32,688
• Total heating pump fault: 26,454
• Return air temperature sensor fault: 5569
• Supply air temperature sensor fault: 536
• Valve position fault: 1

This combined dataset, with a size of 6.8 MB, provides a holistic view of AHU performance and faults. These labels are crucial for training and testing machine learning models aimed at predicting and diagnosing AHU faults. Table 1 presents the statistics, including the mean (SD), smallest value, 25th percentile, median value, 75th percentile, and largest value.

**Table 1:** Description of independent variables.

Independent variables	Description	Unit	Statistics
Set point temperature	The temperature setting for the HVAC system	°C	Mean (SD): 18.861 (2.039) Smallest: 18.0 25th percentile: 18.0 Median: 18.0 75th percentile: 18.0 Largest: 30.0
Return temperature	The temperature of the ventilated air	°C	Mean (SD): 13.342 (5.373) Smallest: −1.63 25th percentile: 9.48 Median: 11.81 75th percentile: 14.96 Largest: 28.13
Supply air temperature	The temperature of the air supplied by the AHU system	°C	Mean (SD): 24.507 (7.467) Smallest: 8.58 25th percentile: 17.54 Median: 26.3 75th percentile: 29.48 Largest: 47.92
Supply fan	The operational status of the supply fan in the AHU system	%	Mean (SD): 0.197 (0.383) Smallest: 0.0 25th percentile: 0.0 Median: 0.0 75th percentile: 0.0 Largest: 1.0
Valve position	The position of the valve in the AHU system	%	Mean (SD): 14.422 (17.3) Smallest: 8.46 25th percentile: 10.0 Median: 10.0 75th percentile: 10.24 Largest: 100.0
Heating supply temperature 1	The temperature of the heating supply at the first measurement point	°C	Mean (SD): 37.671 (10.081) Smallest: 19.57 25th percentile: 33.58 Median: 41.04 75th percentile: 43.24 Largest: 58.84
Heating supply temperature 2	The temperature of the heating supply at the second measurement point	°C	Mean (SD): 36.351 (9.725) Smallest: 18.95 25th percentile: 29.97 Median: 39.75 75th percentile: 42.71 Largest: 54.38
Total heating pump	The total operational status of all heating pumps combined	%	Mean (SD): 1.242 (0.418) Smallest: 0.25 25th percentile: 1.0 Median: 1.0 75th percentile: 1.5 Largest: 2.0
Heating Pump 1	The operational status of the first heating pump.	%	Mean (SD): 0.109 (0.306) Smallest: 0.0 25th percentile: 0.0 Median: 0.0 75th percentile: 0.0 Largest: 1.0
Heating Pump 2	The operational status of the second heating pump	%	Mean (SD): 0.348 (0.462) Smallest: 0.0 25th percentile: 0.0 Median: 0.0 75th percentile: 1.0 Largest: 1.0
Backup heating pump	The operational status of the backup heating pump	%	Mean (SD): 0.785 (0.399) Smallest: 0.0 25th percentile: 1.0 Median: 1.0 75th percentile: 1.0 Largest: 1.0

**Fig. 2:**
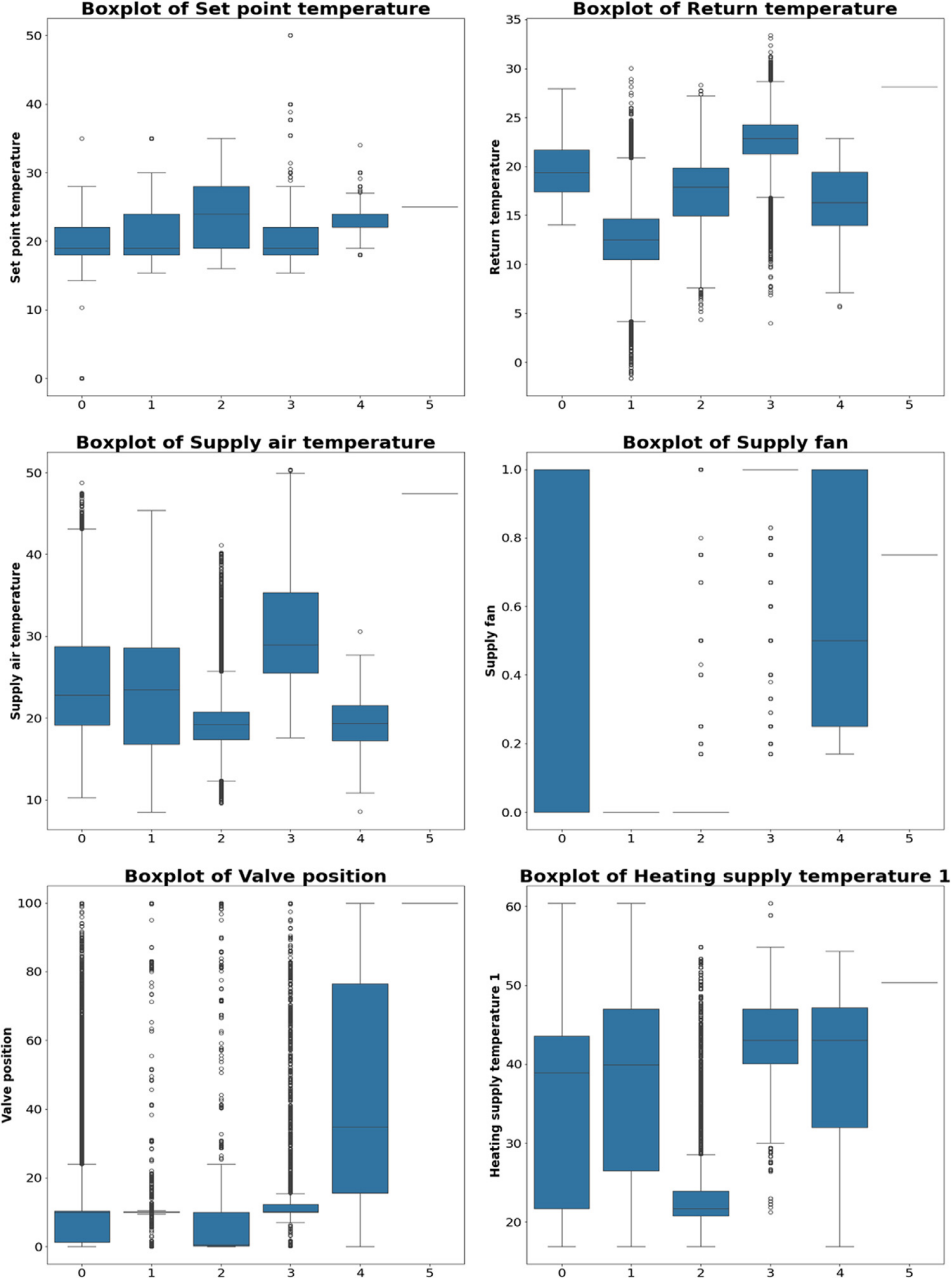
Boxplot1 of each value with labelling.

**Fig. 3:**
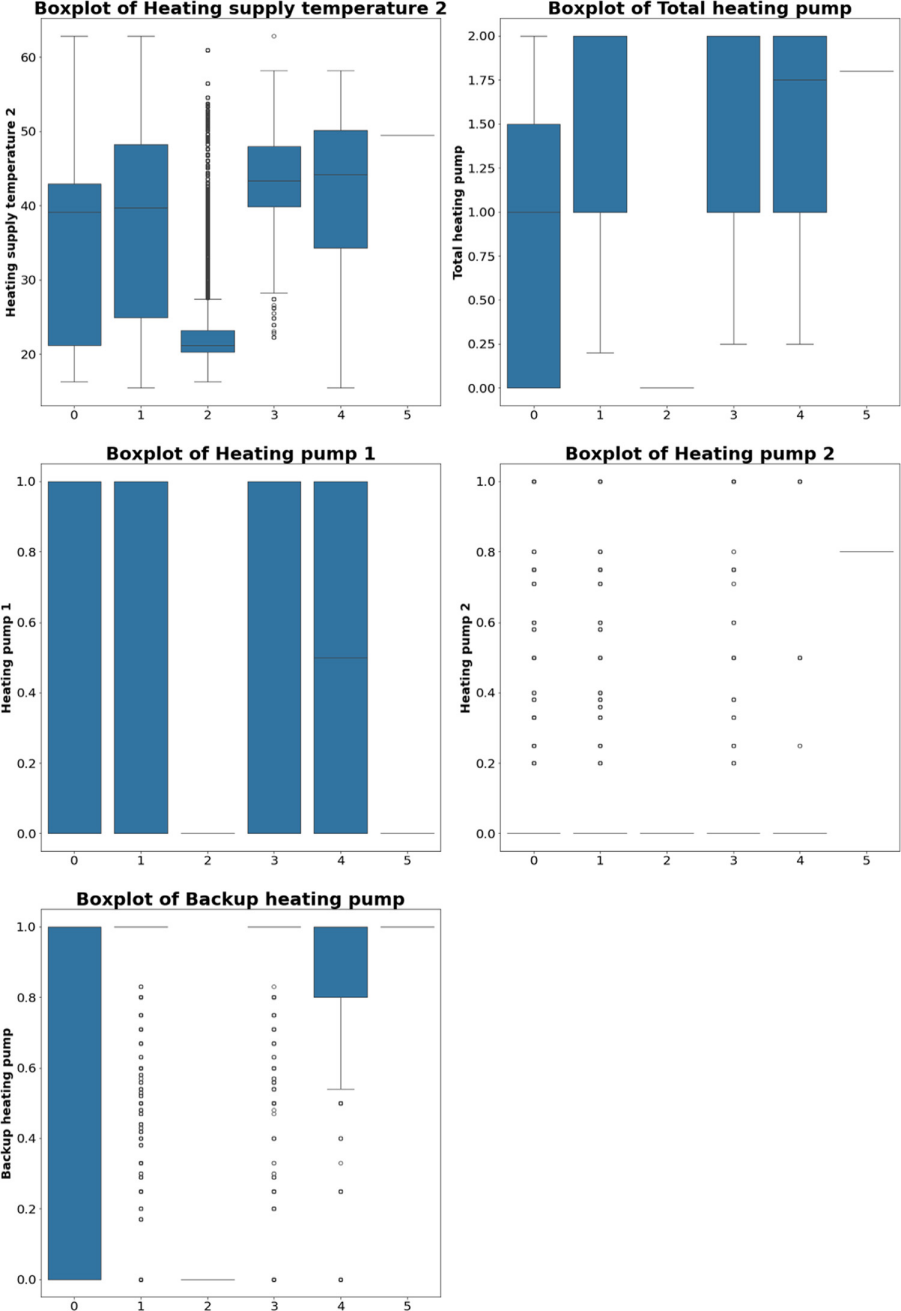
Boxplot2 of each value with labelling.

The box plot analysis reveals significant changes in several AHU parameters under different fault conditions. Fig. 2, Fig. 3 provide the box plot results of each sensor with labeling information. Each label—normal condition, supply fan fault, total heating pump fault, return air temperature sensor fault, supply air temperature sensor fault, and valve position fault—is represented on the x-axis as 1, 2, 3, 4, 5, and 6, respectively, due to limited space.

The set point temperature demonstrates a narrow range of values across all fault types. The median remains consistent regardless of the fault label, indicating stable and well-controlled set point temperatures. This stability makes it a reliable reference point for the system's intended performance. Return temperature shows significant variation across different fault types. Faults like 'Supply fan fault' and 'Return air temperature sensor fault' exhibit wider ranges and higher median values compared to ʻNormal conditionʼ. This suggests that return temperature is highly sensitive to certain faults, making it a critical variable for diagnosis and indicating inefficiencies in the system's air circulation. Supply air temperature exhibits notable differences across fault types. ʻSupply fan faultʼ and ʻTotal heating pump faultʼ have higher median values compared to ʻNormal conditionʼ, highlighting potential problems in air delivery or heating. The increased variance under fault conditions emphasizes the importance of this parameter in fault detection.

The operational status of the supply fan displays distinct patterns across different fault types. ʻSupply fan faultʼ has a low median with a narrow range, as expected. Other fault types also show variability in supply fan status, helping differentiate between normal and faulty operations. The low median in ʻSupply fan faultʼ confirms issues with the fan's operation. Valve position exhibits significant variance across various faults. 'Valve position fault' has higher median values and wider ranges, indicating issues with the valve's operation. While other faults also affect valve position, the impact is less pronounced compared to 'Normal condition'. This highlights its role in regulating flow within the AHU and its importance in diagnosing faults. Both heating supply temperatures show similar trends. Under fault conditions, they display higher medians and greater variances, especially in ʻTotal heating pump faultʼ and ʻSupply air temperature sensor faultʼ. The higher values suggest inefficiencies in the heating system, emphasizing the importance of these parameters in detecting heating-related issues. The operational status of the total heating pump varies significantly across fault conditions.ʻTotal heating pump faultʼ shows a distinct low median, indicating a malfunction. While other faults also exhibit some variance, it is less pronounced. The low median underscores the critical role of this parameter in maintaining effective heating operations.

Both heating pumps display distinct patterns in fault conditions. Heating-related faults, such as ʻTotal heating pump faultʼ, show higher variability and lower medians compared to ʻNormal conditionʼ. This suggests that the statuses of these pumps are important for diagnosing heating issues and provide valuable insights into the performance of the heating system. The backup heating pump shows less variance compared to the primary heating pumps, but fault conditions still impact its operation. Noticeable differences in median values under fault conditions indicate that the backup heating pump is affected by system issues. Monitoring this pump can provide additional insights into the overall health of the heating system and its ability to handle faults.

**Fig. 4:**
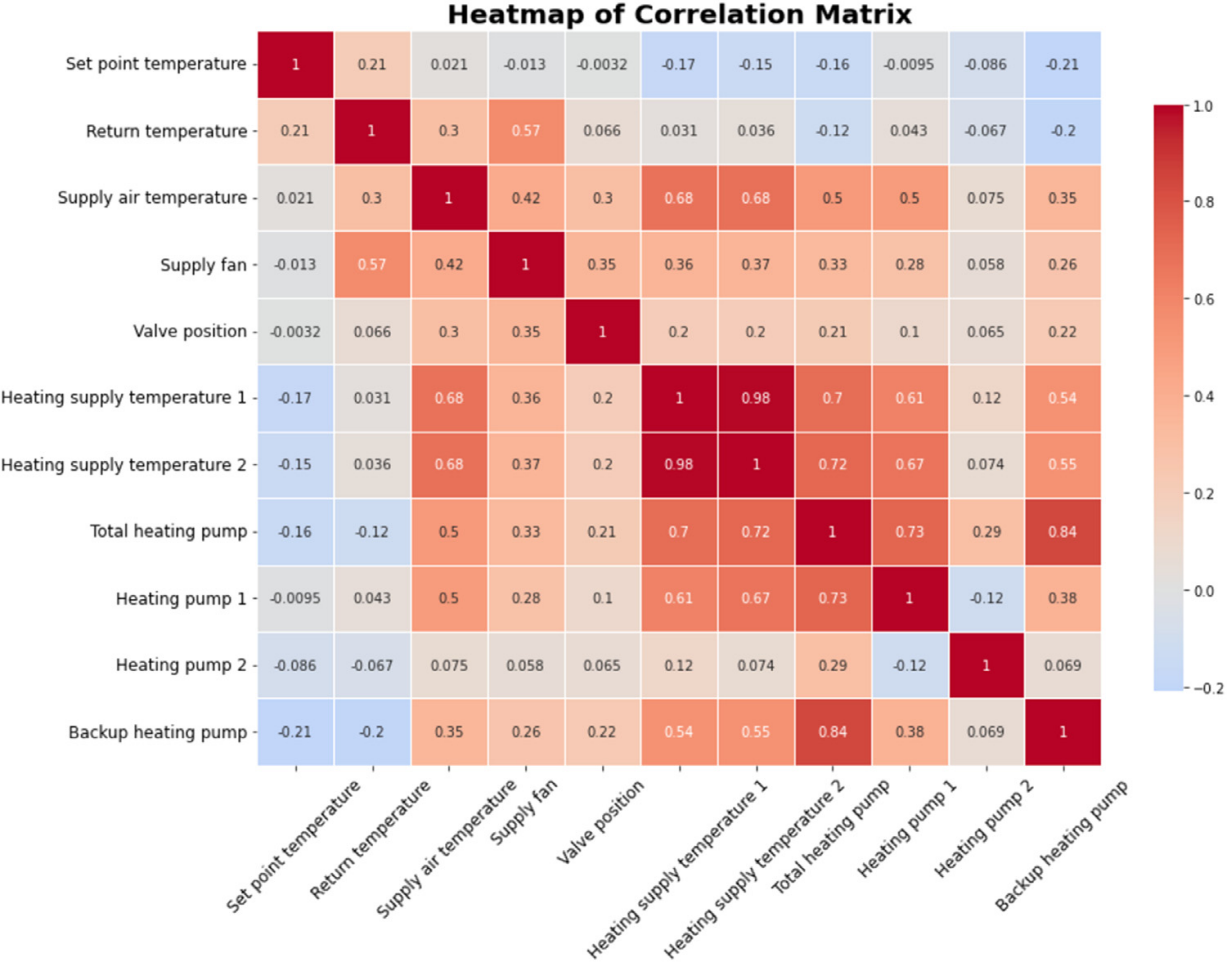
Heatmap of correlation analysis for 11 Variables.

The pearson correlation coefficient was also computed for each pair of variables. This coefficient, ranging from −1 to 1, measures the linear relationship between two variables. A value close to 1 indicates a strong positive correlation, −1 indicates a strong negative correlation, and 0 suggests no linear correlation. A heatmap was generated to visually represent the correlation matrix, which is shown in Fig 4. The heatmap utilized a color gradient to depict the strength of the correlations, with darker shades indicating stronger correlations, whether positive or negative.

The set point temperature exhibited weak positive correlations with most other variables. This stability suggests that it operates relatively independently, not significantly influencing or being influenced by other parameters. Return temperature displayed moderate positive correlations with supply air temperature (0.57) and valve position (0.46). This implies that an increase in return temperature is associated with increases in these variables, indicating a relationship between return air efficiency and system regulation. Supply air temperature showed notable positive correlation with return temperature (0.57) and moderate correlations with heating supply temperatures. This suggests that supply air temperature is significantly influenced by the overall heating efficiency and return air conditions.

The operational status of the supply fan showed weak correlations with other variables, indicating that the supply fan operates relatively independently from other measured parameters, underscoring its distinct role within the system. Valve position exhibited significant variance across various faults, with faults like 'Valve position fault' having higher median values and wider ranges. This points to issues with the valveʼs operation and underscores its importance in regulating airflow and temperature within the AHU. These two variables of heating supply temperature 1 & 2were highly correlated with each other (0.92), suggesting they measure similar aspects of the heating system. They also showed moderate positive correlations with other heating-related variables, indicating coordinated operation of the heating components.

The operational status of the total heating pump displayed strong positive correlations with Heating Pump 1 (0.74) and Heating Pump 2 (0.60). This suggests interdependence among these components, where the activation of the total heating pump is likely accompanied by the operation of these individual pumps. Both heating pumps were strongly correlated with each other (0.74) and with the total heating pump, indicating synchronized operation within the heating system. The backup heating pump showed moderate correlations with the total heating pump (0.65) and Heating Pump 1 (0.45), reinforcing the interconnected nature of the heating components within the AHU.

The pearson correlation analysis reveals significant linear relationships among the AHU parameters. Strong correlations among heating-related variables suggest coordinated operation, while weaker correlations of the supply fan and set point temperature indicate more independent functioning.

## 4 Experimental Design, Materials and Methods

### 4.1 Dataset construction

AHU specifications vary significantly in large-scale offices, as they are specifically designed to meet the unique requirements of these buildings. Large-scale office buildings often have customized AHU systems tailored for their specific heating, cooling, and ventilation needs, rather than using off-the-shelf AHUs typically used in residential buildings or small offices. The dataset collected from a large-scale office located at 48 Gwacheon-daero 7na-gil, Gwacheon-si, Gyeonggi-do, Republic of Korea, spans an area of 50,966 m². The specifications of the 20 different installed and operated AHUs, including supply air blowers, return air blowers, and heating capacity, each with their specific purposes, are described in Table 2.

**Table 2:** Description of specification of installed AHUs.

Name	Purpose	Supply air blower (m^2^/h)	Return air blower (m^2^/h)	Heating capacity (W)
AHU-101	1F lobby	32,100	29,600	93,847
AHU-102	1F labarotory	25,700	25,200	69,898
AHU-103	1F office	27,900	24,130	170,107
AHU-104	2F office	21,100	9900	179,540
AHU-105	3F office/ labarotory	36,500	20,885	276,342
AHU-107	4F labarotory	28,600	17,240	199,285
AHU-108	4F-5F office	20,900	14,890	104,323
AHU-109	4F-5F labarotory	16,300	8920	129,960
AHU-110	5F labarotory	20,700	12,580	144,932
AHU-111	6F labarotory	19,000	12,900	124,188
AHU-112	6F office	18,900	15,660	84,843
AHU-201	B1F meeting room	18,400	18,400	116,130
AHU-202	B1F lobby	36,300	27,180	229,104
AHU-203	B1F gym	11,300	9960	58,146
AHU-204	B1F restaurant	13,800	7990	83,214
AHU-205	2F office	11,500	9220	69,345
AHU-206	3F office	11,500	9220	69,345
AHU-207	4F office	11,700	9420	70,551
AHU-208	5F office	11,700	9420	70,551
AHU-209	6F office	11,700	9420	70,551

The data was meticulously labeled into six categories: normal condition, supply fan fault, total heating pump fault, return air temperature sensor fault, supply air temperature sensor fault, and valve position fault. The labeling criteria for these categories, detailed in Table 3, are based on predefined standards to ensure each category accurately represents different fault conditions.

**Table 3:** Labeling basis on six types of classification.

Category	Description	Criteria
Normal condition	Standard operating conditions without any faults	All parameters within expected range; no anomalies or irregular patterns
Supply fan fault	Issues related to the HVAC system's fan	Significant deviations in airflow rate, fan speed, or electrical consumption patterns
Total heating pump fault	Issues related to the heating pump	Lower median and higher variance in heating pump status
Return air temperature sensor fault	Malfunctions in the sensors measuring return air temperature	Return temperature readings outside the 25th to 75th percentile range (13.55 °C to 20.2 °C)
Supply air temperature sensor fault	Malfunctions in the sensors measuring supply air temperature	Supply air temperature readings outside the 25th to 75th percentile range (18.21 °C to 27.44 °C)
Valve position fault	Problems with the heating valve operation	Failure to open/close as commanded, delays in response, abnormal range of motion, irregular position readings

We aimed to ensure data consistency and reliability for annotating fault types of AHUs. Two experts, with 25 years and 35 years of experience in designing AHU systems respectively, annotated this data. First, they established the criteria for each label, as described in Table 3. Second, the 35-year expert performed the initial annotation, and the 25-year expert reviewed and verified it.

### 4.2 Application of semi-supervised learning method

In this dataset, the valve position had only one case, so it was excluded due to insufficient data for training and testing. The remaining data was used for the analysis. Labeling data can be insufficient for training a model, and the process is often time-consuming [5]. Many studies recommend using semi-supervised learning to address this issue [6,7]. Given that this dataset is partially labeled, we employed a semi-supervised learning approach. Specifically, 60 % of the data was labeled, 30 % was left unlabeled to leverage both labeled and unlabeled data, and the remaining 10 % was reserved as a test set.

We began by training a base model using an artificial neural network (ANN) solely on the labeled dataset. Our chosen model architecture for this task is a model, designed to effectively capture the complex relationships within the AHU data. The architecture consists of several layers:

1) Input Layer: The input layer has 11 nodes, corresponding to the 11 features from the dataset.
2) Hidden Layers: The model includes three hidden layers: The first hidden layer contains 64 neurons with ReLU (Rectified Linear Unit) activation functions to handle non-linearities.
3) The second hidden layer has 32 neurons, also using ReLU activation. The third hidden layer features 16 neurons with ReLU activation, acting as a robust feature extractor.
4) Output Layer: The output layer comprises 6 neurons, each representing a class (five fault types and one normal condition), with a softmax activation function to output probability distributions over the classes.

The predicted labels, referred to as pseudo-labels, served as preliminary guesses for the unlabeled data. To ensure the accuracy of these pseudo-labels, we only selected predictions that the model made with high confidence. This step was crucial in maintaining the quality of the training data and avoiding the introduction of noise. Next, the high-confidence pseudo-labeled data were combined with the original labeled dataset, creating a more extensive and diverse training set. This augmented dataset was then used to retrain the model. The process of generating pseudo-labels, selecting the most confident predictions, and retraining the model was repeated iteratively. After this self-training process, the final model was evaluated on a separate test set to determine its performance. The iterative incorporation of high-quality pseudo-labeled data proved to be highly effective, significantly enhancing the model's accuracy and robustness. Here are the virtual results of the F1-score for each class and the overall F1-score:

• Normal condition: 92 %
• Supply fan fault: 85 %
• Total heating pump fault: 88 %
• Return air temperature sensor fault: 83 %
• Supply air temperature sensor fault: 80 %

The overall F1-score, considering all classes, was 85.6 %. This indicates a high level of performance in accurately detecting and diagnosing various AHU faults. The improvement in the F1-scores across all classes demonstrates the effectiveness of the semi-supervised learning approach in leveraging unlabeled data to enhance model performance.

## Limitations

There are critical limitations when using this dataset to develop a deep learning model for HVAC fault detection. First, the dataset is significantly imbalanced, with far more “Normal condition” instances than “Supply fan fault,” "“Total heating pump fault,” “Return air temperature sensor fault,” “Supply air temperature sensor fault,” and “Valve position fault” instances. This can cause the model to be biased towards predicting the majority class, resulting in poor fault detection. To address this, techniques such as oversampling (e.g., SMOTE) can increase minority class samples, while undersampling can reduce majority class samples. Balanced batching ensures equal representation in each training batch, and class weighting adjusts the loss function to better handle minority class misclassifications [8,9].

In addition, sensor and measurement errors, due to malfunctions or calibration issues, introduce noise into the dataset, obscuring true patterns necessary for effective learning. Mitigation strategies include data cleaning to detect and correct erroneous readings, using multiple sensors for cross-verification, regular sensor calibration and maintenance, and deploying real-time monitoring systems with error detection algorithms. By addressing these limitations, the dataset's quality improves, enhancing the deep learning model's performance and reliability in AHU fault detection. In the current dataset, we have constructed a six-month dataset, but a one-year dataset will be publicly available within eight months. This latter dataset can be more meaningful in several ways. For example, the seasonal effectiveness of AHU faults can be investigated. Additionally, the current dataset includes only heating system faults. In the future dataset, cooling system faults will also be included.

## Ethics Statement

Not applicable.

## CRediT authorship contribution statement

**Seunghyeon Wang:** Conceptualization, Methodology, Writing – original draft, Writing – review & editing. **Ikchul Eum:** Conceptualization, Writing – review & editing. **Sangkyun Park:** Methodology, Investigation, Formal analysis. **Jaejun Kim:** Conceptualization, Methodology, Writing – review & editing.

## Data Availability

A semi-labelled dataset for fault detection in air handling units from a large-scale office (Original data) (Figshare)
